# Colorimetric Analysis and Determination of Histamine in Samples of Yellowfin Tuna (*Thunnus albacares*) Marketed in Sardinia (Italy) by a Combination of Rapid Screening Methods and LC-MS/MS

**DOI:** 10.3390/foods11050639

**Published:** 2022-02-22

**Authors:** Giovanni Luigi Pais, Domenico Meloni, Alessandro Graziano Mudadu, Luigi Crobu, Alessandro Pulina, Giannina Chessa

**Affiliations:** 1 Department of Veterinary Medicine, University of Sassari, Via Vienna 2, 07100 Sassari, Italy; glpais@yahoo.it (G.L.P.); boesislac@hotmail.it (L.C.); 2 Veterinary Public Health Institute of Sardinia, Via Duca degli Abruzzi 8, 07100 Sassari, Italy; alessandro.mudadu@izs-sardegna.it (A.G.M.); alessandro.pulina@izs-sardegna.it (A.P.); giannina.chessa@izs-sardegna.it (G.C.)

**Keywords:** histamine, yellowfin tuna, food safety, screening, official control

## Abstract

The consumption of fishery products has been steadily increasing in recent decades. Among the quantitatively more important species, the yellowfin tuna (*Thunnus albacares*), is one of the main at-risk species as regards the possibility to present important levels of histamine and to be associated with the so-called “Scombroid Fish Poisoning”. The main aim of the present study was to evaluate the colorimetric parameters, the occurrence, and the quantification of histamine contamination in yellowfin tuna samples marketed in Sardinia (Italy) by a combination of rapid screening and official control methods. A total of 20 samples of yellowfin tuna loins collected from large retailers, fishmongers and local markets were analyzed for the qualitative and quantitative determination of histamine by the lateral flow test HistaSure™ Fish Rapid Test and LC-MS/MS, respectively. Moreover, all the samples were examined to assess the conformity with the EU rules on labelling and subjected to colorimetric analysis according to the CIE-L*a*b* standard. Visual inspection of yellowfin tuna labels highlighted a 30% of non-compliances. A significant (*p* < 0.05) difference was reported for brightness (*L* *), redness (*a* *), and yellowness (*b* *). The results of histamine occurrence agreed with the food safety criteria (<100 mg/kg) laid down in EC Regulation 2073/2005 in the 95% and in the 90% of the samples with the rapid screening methods and LC-MS/MS, respectively. A highly significant sessional variation (*p* < 0.00001) was pointed out. Moreover, the two methods showed an agreement rate of 85%. The results of the present study confirmed the utility of lateral flow tests for the fast qualitative determination of histamine in yellowfin tuna. Rapid screening test should be strengthened by comparison with the official method especially in case of uncertain or positive results.

## 1. Introduction

The consumption of fishery products has been steadily increasing in the European Union (EU) in recent decades: EU is the largest single market for imported fish and fishery products, representing 34% of total world imports [1]. The fishery products imported in Europe come from more than 120 countries worldwide and the EU puts high attention on quality, fishing, processing, and traceability along the supply chain [2,3,4,5,6,7,8,9,10,11]. Among the quantitatively more important species consumed in the EU, there is the yellowfin tuna (*Thunnus albacares*), a large pelagic fish that prevail in the tropics and subtropics [12]. In its natural state at the distribution stage, yellowfin tuna should be brown in colour. In the EU market, where it is forbidden to use chemicals to colour foods, yellowfin tuna available for sale in fish shops will look brown. One of the main sanitary frauds connected to the marketing of yellowfin tuna in the EU, is the fraudulent use of carbon monoxide (CO), where yellowfin tuna is coloured bright cherry red to look as everybody imagines fresh tuna to look. Since 2003, the presence of CO in yellowfin tuna from South-East Asian countries and from some Member States, has been repeatedly notified through the Rapid Alert System for Food and Feed (RASFF) [13]. In 2018, 79 people were arrested by Europol in *“Operation Tarantello”* for unauthorised treatment of tuna to promote a colour change [14]. CO is a colourless, odourless, and tasteless gas, toxic by inhalation but not by ingestion, injected into yellowfin tuna to counter the oxidation of the meat. CO reacts with the oxy-myoglobin to form Carboxymioglobin, a very stable and oxidation-resistant complex capable of giving muscle tissue a bright cherry red colour for several days. This very inviting colour it may not correspond to the real freshness of the fish and may mask bacterial spoilage. In histidine-rich fish such as yellowfin tuna, it may hide an excessive amount of histamine [13]. Yellowfin tuna is one of the main at-risk species in terms of presence of histamine at high levels: they present a very long shelf-life, are prepared with temperature variations that can support bacterial proliferation by Gram—(*Morganella*, *Klebsiella*, *Proteus*, *E. coli* and *Hafnia*) and metabolism of histidine-by-histidine decarboxylase decomposition [15]. High levels of histamine may be connected with the so-called “scombroid fish poisoning” [16,17], one of the most important seafood-borne disease worldwide [18,19]. In 2017, 105 cases of scombroid poisoning presented with mild episodes after consuming yellowfin tuna loins from Spain were reported in the EU, and out of these, 11 in Italy. Yellowfin tuna had been frozen and later thawed and sold as fresh vacuum-packed tuna [20]. Histamine was the only biological contaminant reported by the RASFF in the EU in 2020 [21]. Altogether, 14 alerts were associated to fish and products thereof mainly from Vietnam (n.6). In the EU, the EC Regulation 2073/2005 [22] reported the level of histamine in fishery products from fish species presenting high amounts of histidine (*Scombridae*, *Clupeidae*, *Engraulidae*, *Coryfenidae*, *Pomatomidae*, *Scombresocidae*) with acceptable quality (≤100 mg/kg), marginal quality (between 100 and 200 mg/kg) and inacceptable quality (≥200 mg/kg). Due to the high-resolution power, sensitivity, flexibility, and reproducibility amongst all, the use of high-performance liquid chromatography (HPLC) is the analytical reference method in the EU [23,24]. Several alternative techniques have been previously described for the quantification of histamine [19,25,26,27,28,29,30,31,32] However, the high technical skills, cost and time required are the most important limits of these techniques. Therefore, sensitive, and rapid cost-effective methods to detect histamine have been developed [33] for Hazard Analysis Critical Control Points (HACCP) purposes in the fishery sector [34]. Commercial immunoassay tests for histamine qualitative determination are commonly accepted due to the ease of use and limited time requisites [34,35]. There is very little information about the histamine contamination in yellowfin tuna marketed in Italy, regarding the post-capture handling and marketing malpractices. Therefore, the main aim of the present study was to evaluate the respect of the food safety criteria for histamine provided by EC Regulation 2073/2005 [22] in yellowfin tuna samples marketed in Sardinia (Italy) by a combination of rapid screening and official control methods. The specific objectives were the following: (a) evaluation of the provisions of the Common Organisation of the Markets of Fishery and Aquaculture Products (CMO) in terms of labelling [36,37]; (b) colorimetric analysis according to the CIE-L*a*b* standard [38]; (c) qualitative determination of histamine by fast-track lateral flow tests (HistaSure™ Fish Rapid Test, LDN, Nordhorn, Germany); (d) quantitative determination of histamine by official control LC-MS/MS method; (e) comparison of the correspondence between the rapid screening methods and the official control methods.

## 2. Materials and Methods

### 2.1. Collection of the Samples and Evaluation of European Union Labelling

From October 2020 to February 2021, a total of 4 sampling visits (1–4) were scheduled in several market types (large retailers, fishmongers, and local fish markets) situated in the municipality of Sassari (Sardinia, Italy). A total of 20 samples (5 for each sampling day) of yellowfin tuna (*T. albacares*) loins were sampled randomly: in detail, 10 samples from large retailers, 5 samples from local fish markets and 5 samples from fish mongers. According to the availability of yellowfin tuna loins in the considered period, a few selling places were sampled several times. The label data of the yellowfin tuna samples were visually assessed to verify compliance with the rules of the Common Organization of the Markets in Fishery and Aquaculture Products (CMO) on the labelling and marketing of fish products [36,37]. All the samples were forwarded to the Veterinary Medicine Department at the University of Sassari (Italy) and were stored frozen before analysis. Samples were thawed in refrigerator to be subsequently subjected to colorimetric analysis, qualitative and quantitative determination of histamine.

### 2.2. Colorimetric Analysis

Colorimetric analysis was carried out with a digital Spectrophotometer Konica Minolta C508i (Konica Minolta Business Solutions Spa, Milan, Italy) according to the CIE L*a*b* system [38], standard illuminant D65, and 10° standard observer specular component included [39]. Each tuna loin was assessed in triplicate and the mean value ± standard deviation (s.d.) was used in statistic data elaboration.

### 2.3. Qualitative Determination of Histamine

Qualitative determination of histamine was carried out by the lateral flow test HistaSure™ Fish Rapid Test (LDN). To obtain results easily comparable with the food safety criteria laid down in EC Regulation 2073/2005 [22], the cut-off [40] has been set to 100 mg/kg histamine according to Crobu et al., 2021 [35]. Briefly, samples have been prepared for the test procedure according to the AOAC Official Method 937.07 [41]: 10 g of each tuna sample were added to 490 mL distilled water and homogenized for 1–2 min in a lab blender (Koenich, Munich, Germany). An aliquot of 100 μL of the filtered homogenate was then pipetted into the Acylation Buffer Vials and incubated for 5 min at room temperature. Another aliquot of 100 μL of the acylated samples were transferred into the Running Buffer Vials before adding the Lateral Flow Device and incubating for 5 min. The Lateral Flow Device was then removed to visually read the results within 5 min. A negative control represented by 100 μL of distilled water was included in each sampling session. The results have been evaluated according to the HistaSure™ Fish Rapid Test (LDN) official protocol [40].

### 2.4. Quantitative Determination of Histamine

The quantification of histamine was carried out at the Laboratory of Environmental Chemistry and Toxicology of the Veterinary Public Health Institute of Sardinia in Sassari using a validated method LC-MS/MS fit for purpose requirements of EC Regulation 2073/2005 [22]. Briefly, 10.0 g of homogenized sample were weighed in 250 mL centrifuge containers and added to 190 mL of ultrapure water, stirred for 1 min and left to rest for 5 min (this step was repeated 3 times). Five mL of extract were withdrawn using a syringe and filtered through a Whatman glass microfiber filter (Maidstone, UK) inside a 10 mL tube. Subsequently, 100 μL were transferred into a 5 mL volumetric flask, added with 50 μL of Histamine D4 solution (Internal Standard) and adjusted to the desired volume with mobile phase A (Table 1). One mL of extract was filtered directly into an autosampler vial to proceed with instrumental analysis. The quantification of histamine was carried out through a UPLC Acquity I Class chromatography system coupled with Micromass Quattro Premier XE triple quadrupole mass spectrometer with electrospray ionization source (ESI) (Waters, Milford, CT, USA) by using an Acquity BEH HILIC 2.1 X chromatographic column, 100 mm, 1.7 μm (Waters) with a VanGuard BEH HILIC 1.7 μm pre-column. Chromatography involved a gradient elution with the use of 10 mM ammonium formate in 95:5 acetonitrile-water (mobile phase A) and 20 mM ammonium formate (pH 3) in ultra-pure water (mobile phase B), according to Table 1. The mass analyzer operated in positive polarity with a capillary voltage set at 0.40 kV. Nitrogen was used as nebulization and desolvation gas at a flow of 50 and 900 L/h, respectively. The source temperature was maintained at 120 °C, the solvation temperature at 350 °C. The analysis was conducted in MRM (Multiple Reaction Monitoring) mode, using the transitions and acquisition parameters reported in Table 2. The method performances were established during validation studies. Parameters obtained are summarized in Table 3. The quality assurance and control of data were achieved using spiked materials and by checking that analysis complied the validation parameters method. Samples were analyzed in triplicate and the mean value ± s.d. was used in statistic data elaboration.

### 2.5. Statistical Analysis

The differences in brightness (L*), redness (a*) and yellowness (b*) between the yellowfin tuna loins and in the concentration of histamine in relation to the sampling session and to the market type, were compared and analyzed with a one-way ANOVA [42]. Moreover, a multiple pairwise comparison between the means of groups through a Tukey HSD (honestly significant differences) post-hoc test was carried out [42]. The results were considered statistically significant when *p* < 0.05.

## 3. Results and Discussion

### 3.1. Collection of the Samples and Evaluation of European Union Labelling

The visual inspection of the yellowfin tuna labels (Table 4) highlighted a high rate of non-compliances [36,37]. The 70% of the labels showed *T. albacares* as a scientific denomination and yellowfin tuna as trade name. The production method and the category of the fishing gear were reported on 55% of the labels. Purse seine has been reported as the main fishing method. Yellowfin tuna caught with large-scale purse seines cannot immediately be handled after catching, with significant delay before cooling and subsequent freezing and storing [43,44]. In case of prolonged delays, post-mortal bacterial spoilage and accumulation of histamine is very frequent [45]. The fishing area (mainly Indian and Pacific Oceans) was indicated in 55% of the labels. Only 40% of the labels indicated previous defrosting. According to previous studies [46,47], labelling was more accurate in large retailers than in local fish markets and fishmongers. In general, the smaller was the market type the more incomplete was the information [2]. The low rate of non-compliances found in large retailers should be linked to the accuracy in the information transmission along this marketing circuit than the other market types [2], to the applied procedures for the referencing of the suppliers and to the specific training programs of the market staff in charge [48]. As previously reported, not detailed labelling or mislabeling may cause consumers’ misunderstanding [2,11,46,47,49,50,51,52,53,54].

### 3.2. Colorimetric Analysis

The results of the colorimetric analysis performed on the yellowfin tuna samples are shown in Table 5. The average values (± s.d.) of brightness (*L* *), redness (*a* *), and yellowness (*b* *) calculated according to the reference standard CIE-L * a * b * [38], were the following: *L* * = 58.90 ± 3.20; *a* * = 8.67 ± 1.79; *b* * = 8.38 ± 1.44. A significant (*p* < 0.05) difference was reported for the colorimetric parameters (Table 6).

### 3.3. Qualitative Determination of Histamine

The qualitative determination of histamine performed by HistaSure™ Fish Rapid Test (LDN) highlighted that 95% of the samples were always <100 mg/kg and agreed with the EU food safety criteria [22]. Only one sample (2C) showed levels >100 mg/kg (Table 7).

### 3.4. Quantitative Determination of Histamine

The quantitative determination of histamine carried out using the official LC-MS/MS method showed that 90% of the samples were <100 mg/kg in accordance with the EU food safety criteria [22]. Two samples (1B and 1E) showed levels >100 mg/kg (Table 7). ANOVA and Tukey test showed a highly significant sessional variation (*p* < 0.00001) of histamine concentration in examined yellowfin tuna samples (Table 8). Previous studies carried out in tuna samples collected at the retail stage showed histamine levels of 0.45–83.73 mg/kg [18]. Histamine was found at mean levels of 8.9 mg/kg in 83.3% of tuna samples in Spain, while a sample collected from the Netherlands showed high histamine levels (1439 mg/kg) exceeding the EU food safety criteria [18,55,56,57].

**Table 4:** Labelling of yellowfin tuna samples included in the study.

Mandatory Information	Samples																				Total (%)
	1A	1B	1C	1D	1E	2A	2B	2C	2D	2E	3A	3B	3C	3D	3E	4A	4B	4C	4D	4E	
Market type	Local fish market	Local fish market	Fishmonger	Fishmonger	Fishmonger	Large Retailer	Local fish market	Fishmonger	Large Retailer	Large Retailer	Large Retailer	Local fish market	Large Retailer	Large Retailer	Large Retailer	Large Retailer	Local fish market	Fishmonger	Large Retailer	Large Retailer	
Scientific name *(Thunnus albacares)*	yes	yes	-	-	-	yes	yes	-	yes	yes	yes	yes	yes	yes	yes	-	-	yes	yes	Yes	70
Commercial designation (Yellowfin tuna)	yes	yes	-	-	-	yes	yes	-	yes	yes	yes	yes	yes	yes	yes	-	-	yes	yes	yes	70
Production method	-	-	-	-	-	yes	-		yes	yes	yes	yes	yes	yes	yes	-	-	yes	yes	yes	55
Fishing area	-	--	-	-	-	yes	-	-	yes	yes	yes	yes	yes	yes	yes	-	-	yes	yes	yes	55
Fishing gear	-	-	-	-	-	yes	-	-	yes	yes	yes	yes	yes	yes	yes	-	-	yes	yes	yes	55
Defrosting	-	-	-	-	-	yes	-	-	yes	yes	yes	-	-	yes	yes	-	-	-	yes	yes	40

**Table 5:** Colorimetric parameters (mean ± s. d.) of yellowfin tuna samples included in the study.

Colorimetric Parameters	Samples (Mean* ± s.d.)																				Mean Values ± s.d.
	1A	1B	1C	1D	1E	2A	2B	2C	2D	2E	3A	3B	3C	3D	3E	4A	4B	4C	4D	4E	
(*L* *) brightness	59.53 ± 1.71	58.57 ± 0.47	61.56 ± 4.29	51.42 ± 3.50	55.17 ± 3.67	61 ± 3.28	48.77 ± 5.96	52.55 ± 3.52	59.79 ± 2.03	62.15 ± 4.97	65.95 ± 3.38	57.47 ± 6.52	65.02 ± 1.31	67.5 ± 0.70	62.55 ± 1.24	54.23 ± 3.90	62.99 ± 3.91	57.43 ± 1.65	57.99 ± 2.50	56.48 ± 5.57	58.90 ± 3.20
(*a* *) redness	7.52 ± 0.85	11.97 ± 2.57	9.55 ± 0.53	10.33 ± 1.70	4.15 ± 0.23	4.65 ± 0.53	12.79 ± 1.21	13.13 ± 4.90	3.80 ± 1.24	7.60 ± 2.63	6.88 ± 0.53	9.23 ± 3.75	10.22 ± 1.60	7.73 ± 1.02	4.50 ± 0.38	8.63 ± 1.99	13.76 ± 3.44	11.04 ± 2.78	8.87 ± 1.94	7.03 ± 2.08	8.67 ± 1.79
(*b* *) yellowness	5.42 ± 0.63	9.37 ± 1.99	8.36 ± 1.04	7.87 ± 2.11	9.24 ± 1.54	8.33 ± 0.19	13.17 ± 1.96	10.38 ± 3.47	7.71 ± 0.55	8.56 ± 1.96	6.73 ± 0.84	5.76 ± 2.70	8.59 ± 1.15	6.04 ± 1.08	6.77 ± 0.58	12.31 ± 2.27	5.82 ± 0.45	6.96 ± 1.61	9.83 ± 0.65	10.47 ± 2.09	8.38 ± 1.44

* Each sample was measured in triplicate.

**Table 6:** Differences among the colorimetric parameters in yellowfin tuna samples from ANOVA and the Tukey test.

Sample	1A	1B	1C	1D	1E	2A	2B	2C	2D	2E	3A	3B	3C	3D	3E	4A	4B	4C	4D	4E
1A							*b* ***									b**				
1B					*a* **	*a* *			*a* **						*a* *					
1C							*L* *													
1D							*b* *				*L* **		*L* **	*L* ***			*L* *			
1E							*a* **	*a* **						L*			*a* ***	*a* *		
2A							*L* * *a* **	*a* **									a**			
2B									*a* ** *b* *	*L* **	*L* *** *b* **	*b* ***	*L* ***	*L* **** *b* ***	L ** a ** b **		L ** b ***	*b* **		
2C									*a* ***		*L* **		*L* *	*L* **	*a* **					
2D																	*a* ***	*a* *		
2E																				
3A																*L* * *b* *	a*			
3B																*b* **				
3C																				
3D																L ** b **				
3E																*b* *	*a* ***			
4A																	*b* **	*b* *		
4B																				*a* *
4C																				
4D																				
4E																				

*L* = brightness; *a* = redness; *b* = yellowness; **** *p* < 0.0001; *** *p* < 0.001; ** *p* < 0.01; * *p* < 0.05.

**Table 7:** Qualitative and quantitative determination of histamine in of yellowfin tuna samples included in the study.

Samples (Mean ± s.d.)																				
Market Type	Local Fish Market	Local Fish Market	Fishmonger	Fishmonger	Fishmonger	Large Retailer	Local Fish Market	Fishmonger	Large Retailer	Large Retailer	Large Retailer	Local Fish Market	Large Retailer	Large Retailer	Large Retailer	Large Retailer	Local Fish Market	Fishmonger	Large Retailer	Large Retailer
Histamine determination	1A	1B	1C	1D	1E	2A	2B	2C	2D	2E	3A	3B	3C	3D	3E	4A	4B	4C	4D	4E
Qualitative (HistaSure ™ Fish Rapid Test)	<100 mg/kg	<100 mg/kg	<100 mg/kg	<100 mg/kg	<100 mg/kg	<100 mg/kg	<100 mg/kg	>100 mg/kg	<100 mg/kg	<100 mg/kg	<100 mg/kg	<100 mg/kg	<100 mg/kg	<100 mg/kg	<100 mg/kg	<100 mg/kg	<100 mg/kg	<100 mg/kg	<100 mg/kg	<100 mg/kg
Quantitative (LC/MS-MS) (mean ± s.d.)	10.35 ± 0.02 mg/kg	332.24 ± 0.01 mg/kg	0.73 ± 0.02 mg/kg	0.64 ± 0.02 mg/kg	263.19 ± 0.02 mg/kg	24.38 ± 0.02 mg/kg	0.97 ± 0.02 mg/kg	73.50 ± 0.01 mg/kg	0.49 ± 0.02 mg/kg	0.41 ± 0.02 mg/kg	0.30 ± 0.02 mg/kg	0.67 ± 0.01 mg/kg	14.22 ± 0.02 mg/kg	0.36 ± 0.02 mg/kg	5.11 ± 0.02 mg/kg	0.34 ± 0.02 mg/kg	0.91 ± 0.02 mg/kg	0.30 ± 0.02 mg/kg	25.09 ± 0.02 mg/kg	9.36 ± 0.02 mg/kg

**Table 8:** Differences among the concentration of histamine in yellowfin tuna samples from ANOVA and the Tukey test.

Sample	1A	1B	1C	1D	1E	2A	2B	2C	2D	2E	3A	3B	3C	3D	3E	4A	4B	4C	4D	4E
1A		*****	*****	*****	*****	*****	*****	*****	*****	*****	*****	*****	*****	*****	*****	*****	*****	*****	*****	*****
1B			*****	*****	*****	*****	*****	*****	*****	*****	*****	*****	*****	*****	*****	*****	*****	*****	*****	*****
1C				***	*****	*****	*****	*****	*****	*****	*****	*	*****	*****	*****	*****	*****	*****	*****	*****
1D					*****	*****	*****	*****	*****	*****	*****		*****	*****	*****	*****	*****	*****	*****	*****
1E						*****	*****	*****	*****	*****	*****	*****	*****	*****	*****	*****	*****	*****	*****	*****
2A							*****	*****	*****	*****	*****	*****	*****	*****	*****	*****	*****	*****	*****	*****
2B								*****	*****	*****	*****	*****	*****	*****	*****	*****		*****	*****	*****
2C									*****	*****	*****	*****	*****	*****	*****	*****	*****	*****	*****	*****
2D										***	*****	*****	*****	*****	*****	*****	*****	*****	*****	*****
2E											*****	*****	*****		*****	**	*****	*****	*****	*****
3A												*****	*****	*	*****		*****		*****	*****
3B													*****	*****	*****	*****	*****	*****	*****	*****
3C														*****	*****	*****	*****	*****	*****	*****
3D															*****		*****	*	*****	*****
3E																*****	*****	*****	*****	*****
4A																	*****		*****	*****
4B																		*****	*****	*****
4C																			*****	*****
4D																				*****
4E																				

***** *p* < 0.00001; *** *p* < 0.001; ** *p* < 0.01; * *p* < 0.05.

## 4. Conclusions

The vacuum-packed yellowfin tuna loins are at the top of the risk as regards the presence of histamine in fish products. Moreover, the EU has banned CO treatment in fish: in the treated products, the colour may mask the deterioration associated with potential risk of scombroid syndrome [13] and “suspect” samples for CO should also be sampled for histamine. The determination of the colorimetric parameters was intended to highlight any correlations between high levels of histamine and a possible use of CO through a quick and easy-to-use colorimetric test. Although many of the yellowfin tuna samples included in our study showed a bright red cherry colour with significant (*p* < 0.05) differences, even in presence of “suspicious” staining, the comparison between the colorimetric parameters and the results of the quantitative determination of the histamine level, did not show any substantial correlation. Marketing practices may significantly influence the levels of histamine in yellowfin tuna: the highest levels were observed in samples collected from small fishmongers and local markets, where were displayed for more than 8 h at temperatures >5 °C considerably compromising the safety and quality of yellowfin tuna [57]. According to previous studies [34] which reported a good overlap of the results obtained from HistaSure™ Fish Rapid Test (LDN) with HPLC results, this study showed an agreement of 85% between the results obtained by rapid screening methods and those obtained by the official LC-MS/MS method. However, 10% of false negative results and 5% of false positive results obtained in this study highlighted that lateral flow tests must be reinforced by official methods if doubtful or positive results are achieved [50].

## Figures and Tables

**Table 1:** Gradient elution of the mobile phases A (acetonitrile-water) and B (ultra-pure water) with 10 mM and 20 mM ammonium formate.

Time (min)	%A	%B	Flow (mL/min)	Volume of Injection (µL)
0.00	90	10	0.6	5
0.10	90	10		
3.00	70	30		
4.00	50	50		
4.50	50	50		
4.51	50	50		

**Table 2:** Transitions and acquisition parameters used in MRM (Multiple Reaction Monitoring) mode.

Analysis	Parent (m/z)	Daughter (m/z)	Dwell Time (s)	Cone Voltage (V)	Collision Energy (V)
Histamine	111.9	67.9	0.025	50	22
	111.9	94.9 Q	0.025	50	14
Histamine D4	116.10	99.10	0.025	50	14

**Table 3:** LC-MS/MS validation Parameters.

Analytical Parameters	Value
Concentration Interval	5–400 mg/kg
Linearity (R^2^)	>0.9995
Recoveries	98–103%
Limit of quantification (LOQ)	1.00 mg/kg
Intra-day precision (repeatability)	0.7–3%
Inter-day precision (within-lab reproducibility)	3–4%

## Data Availability

Not applicable.
